# High-quality metagenome-assembled genomes of carbon-degrading, sulfate-reducing, and sulfur-oxidizing Acidobacteriota from Sub-Antarctic Marion Island soils

**DOI:** 10.1128/mra.00342-26

**Published:** 2026-06-15

**Authors:** Phillip Mawire, Matteo N. J. Gregori, John P. Makumbi, Oliver K. Bezuidt, Thulani P. Makhalanyane

**Affiliations:** 1 Department of Biochemistry, Genetics and Microbiology, University of Pretoria, Hatfield 56410, Pretoria, South Africa; 2 Department of Microbiology, Faculty of Science, Stellenbosch University 26697, Stellenbosch, South Africa; 3 School for Data Science and Computational Thinking, Stellenbosch University 26697, Stellenbosch, South Africa; Montana State University, Bozeman, Montana, USA

**Keywords:** acidobacteriota, biogeochemical cycling, extreme environments, Marion Island, sub-antarctic ecosystems

## Abstract

Here, we present high-quality Acidobacteriota metagenome-assembled genomes (*n* = 20) belonging to understudied lineages (UBA7541 [*n* = 13] and SbA1 [*n* = 7]) from sub-Antarctic soils. Nutrient cycling genes were prevalent in these MAGs, which provide a resource for understanding the ecological role of Acidobacteriota in extreme environments.

## ANNOUNCEMENT

Nutrient availability constrains taxonomic diversity and metabolic capacity in extreme environments (1–3), including the sub-Antarctic Marion Island. In these regions, GC-rich microbes, including members of the phylum Acidobacteriota thrive, with high relative abundances and metabolic diversity (4–6). These taxa play a role in biogeochemical recycling, contributing to carbon, nitrogen, and sulfur turnover (4–6). Despite this ecological importance, the functional roles of Acidobacteriota remain poorly characterized, partly because representative genomic resources are limited (5). Here, we expand these genomic resources by providing insights from understudied Acidobacteriota with capacity for organic matter turnover and nutrient cycling on Marion Island.

Eight composite soil samples were collected from Marion Island’s Fernbrake (*n* = 5) and Fellfield (*n* = 3) habitats in April and May 2023 (Table 1; Sample metadata at https://doi.org/10.6084/m9.figshare.31584037). At each site, surface soil was collected from five spatially distributed points, combined into a homogenized composite sample, and stored at −80°C until processing. Metagenomic DNA was extracted from each composite sample using the PowerSoil DNA Isolation Kit (Qiagen, USA)(7). The Qubit dsDNA Assay kit (Qubit 4 Fluorometer, Thermo Fisher Scientific, USA) (8) was used to assess the concentration of DNA, and the quality was checked via 1% agarose gel electrophoresis (9). Metagenomic libraries were prepared using the Watchmaker Genomics DNA Library Prep Kit (Watchmaker Genomics, USA) (10) and then sequenced on an Illumina NovaSeq X Plus using a 25B flowcell and paired-end 150 bp reads, yielding 100 million reads per direction for each composite sample.

**TABLE 1:** Sample details, genomic characteristics, and assembly statistics of the Acidobacteriota genomes recovered from Fernbrake and Fellfield habitats soils on the Marion Island

Genome ID	Genome accession number	Raw reads accession number	Sample habitat	Order	Family	Completeness (%)	Contamination (%)	Genome size (bp)	GC content (%)	Contig N50
S1.bin.27	SAMN56290323	SRR37370070	Fernbrake	*Candidatus* Acidiferrales	UBA754	96.97	0.72	4,282,981	58.0	172852
S10.bin.49	SAMN56290324	SRR37370072	Fellfield	*Candidatus* Acidiferrales	UBA754	94.55	3.39	3,117,350	58.0	14447
S11.bin.59	SAMN56290325	SRR37370071	Fellfield	Terriglobales	SbA1	91.90	4.52	4,751,962	58.0	19466
S2.bin.35	SAMN56290326	SRR37370069	Fernbrake	Terriglobales		99.95	0.08	3925298	60.0	150147
S2.bin.56	SAMN56290327	SRR37370069	Fernbrake	*Candidatus* Acidiferrales	UBA754	92.67	1.86	4248230	59.0	102101
S2.bin.61	SAMN56290328	SRR37370069	Fernbrake	Terriglobales	SbA1	100.00	0.07	4228233	58.0	240254
S2.bin.68	SAMN56290329	SRR37370069	Fernbrake	*Candidatus* Acidiferrales	UBA754	91.70	0.00	4362302	58.0	202362
S2.bin.92	SAMN56290330	SRR37370069	Fernbrake	*Candidatus* Acidiferrales	UBA754	93.91	0.08	3425196	60.0	101505
S3.bin.13	SAMN56290331	SRR37370068	Fernbrake	*Candidatus* Acidiferrales	UBA754	96.88	0.31	4111988	58.0	263088
S3.bin.137	SAMN56290332	SRR37370068	Fernbrake	Terriglobales	SbA1	94.87	0.32	4786090	58.0	192939
S3.bin.145	SAMN56290333	SRR37370068	Fernbrake	*Candidatus* Acidiferrales	UBA754	94.52	3.08	4660810	59.0	38002
S3.bin.154	SAMN56290334	SRR37370068	Fernbrake	Terriglobales	SbA1	93.29	0.39	4291985	58.0	117526
S3.bin.155	SAMN56290335	SRR37370068	Fernbrake	Terriglobales	SbA1	95.92	0.32	4871286	57.0	156436
S3.bin.46	SAMN56290336	SRR37370068	Fernbrake	Terriglobales	SbA1	99.99	1.19	5539772	57.0	221770
S3.bin.59	SAMN56290337	SRR37370068	Fernbrake	*Candidatus* Acidiferrales	UBA754	94.04	3.65	3602832	60.0	64782
S4.bin.39	SAMN56290338	SRR37370067	Fernbrake	*Candidatus* Acidiferrales	UBA754	96.29	1.12	4323614	58.0	84912
S4.bin.62	SAMN56290339	SRR37370067	Fernbrake	*Candidatus* Acidiferrales	UBA754	94.44	0.62	3582445	61.0	70555
S5.bin.10	SAMN56290340	SRR37370066	Fernbrake	*Candidatus* Acidiferrales	UBA754	96.69	0.13	3226576	64.0	30850
S5.bin.165	SAMN56290341	SRR37370066	Fernbrake	*Candidatus* Acidiferrales	UBA754	96.99	0.27	4065312	62.0	212762
S7.bin.3	SAMN56290342	SRR37370065	Fellfield	*Candidatus* Acidiferrales	UBA754	90.38	0.83	4210590	60.0	31140

Raw DNA reads were quality-checked using FastQC v0.11.7 (11) for per-base quality, GC content, and adapter contamination, followed by trimming of low-quality bases and residual adapters with Trimmomatic v0.39 (12). All the resultant trimmed paired-end reads for each composite sample were separately assembled *de novo* with metaSPAdes v3.15.5 (13), and contigs ≥500 bp were retained. Bowtie2 v2.4.1 (14) and samtools v1.7 (15) were used to map reads back to the assemblies. MetaBAT2 v2.15 (16) was used to bin assembled contigs for each composite sample into metagenome-assembled genomes (MAGs). MAG dereplication was performed within each composite sample using dRep v3.4.3 (17). GTDB-Tk v2.2.6 was used for taxonomic assignments (18) against the GTDB R207 (19). MAG quality was evaluated with CheckM2 v1.1.0 (20) and high-quality MAGs selected for downstream analysis (completeness >90% and contamination <5%). To determine the metabolic potential of the MAGs, the Prodigal module of METABOLIC v4.0 (20) was used to predict open reading frames (ORFs) followed by hmmsearch to annotate proteins against the HMM databases (KEGG KOfam, Pfam, TIGRfam, and custom HMMs) implemented within METABOLIC v4.0(20).

**Fig 1:**
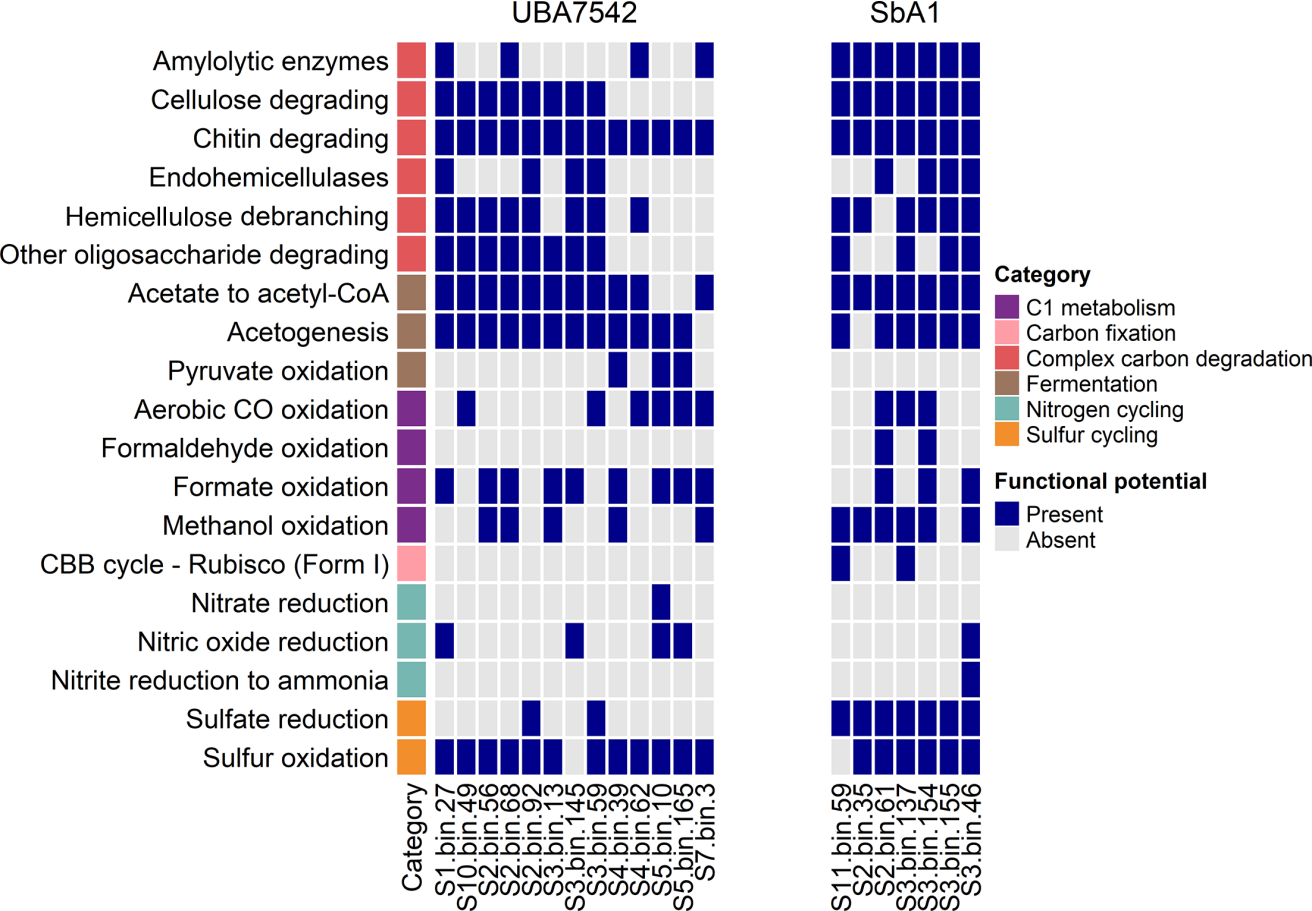
Functional presence–absence heatmap of 20 Acidobacteriota metagenome-assembled genomes (MAGs) recovered from Fernbrake and Fellfield soils on sub-Antarctic Marion Island. The heatmap summarizes 43 curated metabolic functions identified using the METABOLIC v4.0, where a function was scored as present if at least one associated marker protein was detected and absent if none were detected. Complex carbon degradation was the most prevalent category, with chitin degradation detected across all genomes. The full METABOLIC v4.0 FunctionHit output is available at https://doi.org/10.6084/m9.figshare.31584037.

We recovered 20 high-quality Acidobacteriota MAGs from the Fernbrake and Fellfield habitats of Marion Island. All MAGs were assigned to two previously unclassified families (SbA1; *n* = 7 and UBA7542; *n* = 13) affiliated with the orders Terriglobales and Candidatus Acidiferrales, respectively (Table 1). The MAGs harbored genes implicated in organic matter decomposition and nutrient recycling, mainly carbon degradation and sulfur cycling (Fig. 1). These functional signatures imply that these lineages play important roles in biogeochemical cycling within the island’s major terrestrial ecosystems. These genomes constitute a valuable genomic resource for understanding carbon and sulfur cycling in extreme Sub-Antarctic terrestrial ecosystems.

## Data Availability

The genome assemblies for the MAGs, along with the raw reads, have been deposited in NCBI GenBank (BioSample) and the Sequence Read Archive (SRA) under BioProject PRJNA1428722 with the corresponding accession numbers listed in Sample_metadata and MAGs metadata in https://doi.org/10.6084/m9.figshare.31584037.
